# Controlled breathing is associated with lower blood lactate than normobaric hypoxia despite comparable peripheral oxygen saturation during moderate-intensity exercise: an exploratory crossover study

**DOI:** 10.3389/fphys.2026.1916547

**Published:** 2026-09-04

**Authors:** AJ Fisher, Jessie Hirsch, Kaela Walker, Alexander Rothstein, Adrienne Herrenbruck

**Affiliations:** 1 School of Health Sciences, American Public University System, Charles Town, WV, United States; 2 Interdisciplinary Health Sciences, New York Institute of Technology, Old Westbury, NY, United States

**Keywords:** blood lactate, controlled breathing, end-expiratory breath holds, exercise metabolism, intermittent hypoxia, normobaric hypoxia, oxygen desaturation, voluntary hypoventilation

## Abstract

**Background:**

Blood lactate accumulation during exercise has traditionally been associated with reduced oxygen availability and an increased reliance on glycolytic metabolism. However, lactate production is influenced by multiple processes beyond oxygen availability. Previous pilot work demonstrated that a standardized controlled breathing intervention produced intermittent reductions in peripheral oxygen saturation comparable to intermittent hypoxic training, but its effects on blood lactate had not been investigated. This crossover study compared normobaric hypoxia (NH) and controlled breathing (CB). We hypothesized that both interventions would increase blood lactate accumulation relative to control.

**Methods:**

Fifteen healthy adults completed three laboratory visits using a within-subject crossover design. Participants performed an identical 30-minute functional exercise protocol during control (CON), NH (15% inspired oxygen), and CB. Blood lactate was measured at rest, after 10, 20, and 30 minutes of exercise, and after 10 minutes of recovery. Blood lactate AUC, maximum blood lactate, average HR, peripheral oxygen saturation (SpO_2_), and ratings of perceived exertion (RPE) were assessed. Data were analyzed using linear mixed-effects models with Tukey-adjusted pairwise comparisons.

**Results:**

Contrary to hypothesis, NH and CB produced different metabolic responses despite reducing peripheral oxygen saturation. Blood lactate differed among conditions over time (condition × time interaction, p = 0.007), with CB demonstrating lower blood lactate than CON at 10, 20, 30 minutes, and recovery, and lower blood lactate than NH at 20 and 30 minutes. Maximum blood lactate (p = 0.002), blood lactate AUC (p < 0.001), and average HR (p < 0.001) were significantly lower during CB. NH produced greater reductions in peripheral oxygen saturation than CB, although both interventions reduced SpO_2_ relative to CON. RPE did not differ among conditions (p = 0.371).

**Conclusions:**

A standardized controlled breathing intervention was associated with lower blood lactate accumulation, cumulative lactate exposure, peak blood lactate concentration, and average HR despite significant reductions in peripheral oxygen saturation during moderate-intensity exercise. These exploratory findings suggest pulse oximetry-measured peripheral oxygen saturation may not fully predict metabolic responses to exercise. Because underlying mechanisms were not directly assessed, future mechanistic studies using direct measurements of skeletal muscle oxygenation, gas exchange, and metabolic regulation are warranted.

## 1 Introduction

Blood lactate accumulation during exercise has traditionally been attributed to insufficient oxygen availability and an increased reliance on anaerobic glycolysis. Within this classical framework, elevations in blood lactate were interpreted as evidence that oxygen delivery had become insufficient to meet metabolic demand, resulting in greater dependence on non-oxidative ATP production (Wasserman and McIlroy, 1964; Wasserman et al., 1973).

However, contemporary understanding recognizes that lactate production is regulated by a complex interaction of oxygen availability, glycolytic flux, substrate utilization, hormonal responses, autonomic regulation, and lactate clearance rather than by oxygen availability alone (Gladden, 2004; Faude et al., 2009; Brooks, 2018). Lactate is now recognized as an important metabolic intermediate that is continuously produced and utilized during exercise (Gladden, 2004; Brooks, 2020). Blood lactate concentration reflects the dynamic balance between lactate production and clearance and is influenced by numerous physiological processes, including exercise intensity, substrate availability, sympathetic activation, and mitochondrial oxidative capacity (Gladden, 2004; Faude et al., 2009; Brooks, 2018). Accordingly, similar reductions in peripheral oxygen saturation (SpO_2_) should not necessarily be expected to produce similar blood lactate responses, particularly when other physiological factors influencing glycolytic metabolism differ between interventions (Brooks, 2018; Brooks, 2020). Whether similar reductions in peripheral oxygen saturation produce comparable metabolic responses during exercise therefore remains uncertain.

Moreover, the physiological consequences of reduced peripheral oxygen saturation may depend not only on the magnitude of oxygen desaturation but also on its temporal pattern, including whether reductions occur as sustained decreases or as repeated transient desaturation–recovery cycles (Mitchell et al., 2001; Navarrete-Opazo and Mitchell, 2014). Controlled breathing strategies incorporating prolonged exhalation and timed end-expiratory breath holds represent one potential approach for modifying the physiological response to exercise; however, the mechanisms through which these interventions influence metabolic responses remain incompletely understood (Woorons et al., 2020). Alterations in ventilatory patterns influence arterial gas exchange, respiratory mechanics, cardiovascular function, and exercise metabolism through multiple interacting pathways (Woorons et al., 2010; Woorons et al., 2019; Woorons et al., 2020).

Importantly, peripheral oxygen saturation measured by pulse oximetry reflects arterial hemoglobin oxygen saturation and should not be interpreted as a direct measure of skeletal muscle oxygenation, tissue oxygen availability, or cellular hypoxia (Richardson et al., 1995; Jubran, 2015). These variables represent distinct physiological constructs that require separate measurement techniques and were not directly assessed in the present investigation.

The standardized controlled breathing intervention evaluated in the present study was developed from previous pilot work demonstrating that prolonged exhalation combined with timed end-expiratory breath holds produced intermittent reductions in peripheral oxygen saturation during moderate-intensity exercise comparable to those observed during intermittent hypoxic training (Brindisi et al., 2024). However, whether inducing reductions in peripheral oxygen saturation through this standardized breathing strategy would produce metabolic responses similar to those observed during normobaric hypoxic exercise had not been investigated.

Previous studies have reported that voluntary hypoventilation during exercise can induce transient reductions in peripheral oxygen saturation while increasing blood lactate concentrations despite similar external workloads (Woorons et al., 2010; Woorons et al., 2019; Woorons et al., 2020). Likewise, exercise performed under normobaric hypoxic conditions is commonly associated with greater blood lactate accumulation than equivalent exercise performed under normoxic conditions (Millet et al., 2016; Schiffer et al., 2017; Faulhaber et al., 2020). Collectively, these findings, together with our pilot investigation demonstrating that the standardized controlled breathing intervention reduced peripheral oxygen saturation during exercise (Brindisi et al., 2024), formed the basis for our original hypothesis that interventions capable of reducing peripheral oxygen saturation would result in greater blood lactate accumulation during moderate-intensity exercise.

Unlike traditional voluntary hypoventilation protocols, the controlled breathing intervention consisted of a standardized breathing–movement strategy in which inhalation timing, prolonged exhalation, end-expiratory breath holds, and movement execution were intentionally synchronized throughout the exercise session. This intervention was designed to provide a reproducible breathing protocol that could be consistently implemented across participants while inducing intermittent reductions in peripheral oxygen saturation during moderate-intensity exercise.

To provide an appropriate physiological comparator, a normobaric hypoxic intervention was included because it represented an alternative strategy for reducing peripheral oxygen saturation during standardized exercise, allowing direct comparison of two distinct approaches to reducing peripheral oxygen saturation under otherwise similar exercise conditions.

The primary objective of this exploratory crossover study was to compare metabolic, cardiovascular, and oxygenation responses across three experimental conditions: control exercise (CON), normobaric hypoxic exercise (NH), and a standardized controlled breathing intervention (CB). Primary outcomes included blood lactate concentration, peripheral oxygen saturation (SpO_2_), average heart rate, and rating of perceived exertion (RPE). Based on previous evidence that acute normobaric hypoxia reduces peripheral oxygen saturation and is commonly associated with greater blood lactate accumulation during exercise (Millet et al., 2016; Schiffer et al., 2017; Brooks, 2020; Faulhaber et al., 2020), together with our previous pilot study demonstrating that the standardized controlled breathing intervention also reduced peripheral oxygen saturation during standardized exercise (Brindisi et al., 2024), we hypothesized that both interventions would produce greater blood lactate accumulation than the control condition under otherwise standardized exercise conditions.

## 2 Materials and methods

### 2.1 Participants

Healthy adults were recruited from the New York Institute of Technology community through classroom announcements, electronic communications, and word-of-mouth recruitment. Participants were eligible if they were between 18 and 40 years of age, recreationally active, and medically cleared to perform moderate-intensity exercise. Individuals were excluded if they reported a history of cardiovascular, pulmonary, neurological, or metabolic disease; uncontrolled hypertension; pregnancy; musculoskeletal injury limiting exercise participation; previous experience with structured breathing-training programs; or any contraindication to hypoxic exposure or breath-hold exercise.

Prior to participation, all volunteers completed a health history questionnaire and provided written informed consent. The study protocol was approved by the Institutional Review Boards of the New York Institute of Technology and the American Public University System and was conducted in accordance with the ethical principles outlined in the Declaration of Helsinki.

**Figure 1:**
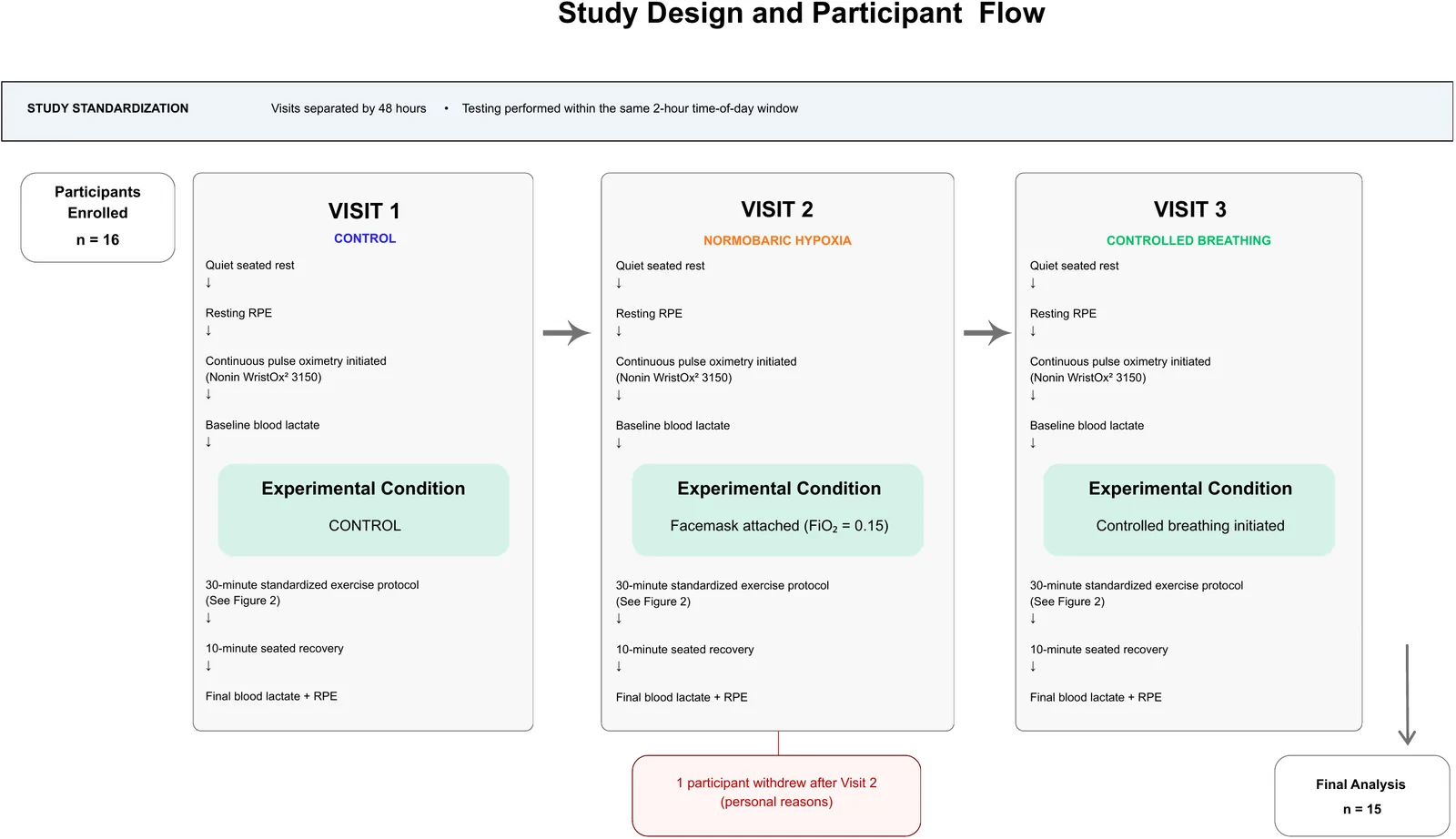
Study design and participant flow. Sixteen participants completed Visits 1 and 2. One participant withdrew for personal reasons after Visit 2, leaving 15 participants for the final repeated-measures analyses. Visits were separated by at least 48 hours and performed within the same 2-hour time-of-day window to minimize potential circadian effects.

Sixteen participants were initially enrolled in the study. One participant voluntarily withdrew after the second laboratory visit and did not complete the controlled breathing condition. Consequently, only participants who completed all three experimental conditions were included in the final analyses, resulting in a final analytical sample of 15 participants (**Figure 1**). Participant characteristics are presented in **Table 1**.

**Table 1:** Participant characteristics.

Variable	Mean ± SD or n
Participants (n)	15
Sex Distribution (F/M)	7/8
Age (years)	23.6 ± 2.0
Height (cm)	167.9 ± 6.9
Weight (kg)	71.8 ± 16.4
Body Mass Index (kg/m^2^)	25.2 ± 4.3

Values are presented as mean ± standard deviation unless otherwise indicated.

Baseline demographic and anthropometric characteristics of participants included in the final analysis (n = 15).

An *a priori* sample size analysis was performed using G*Power (Version 3.1; Heinrich Heine University Düsseldorf, Düsseldorf, Germany). Based on an anticipated moderate effect size (*f* = 0.35), an alpha level of 0.05, statistical power of 0.80, and a repeated-measures study design, the estimated minimum sample size required was 15 participants. To account for potential attrition, 16 participants were recruited.

### 2.2 Study design

This exploratory within-subject crossover study compared physiological responses during three experimental exercise conditions: (1) control exercise performed under normoxic conditions (CON), (2) standardized exercise performed under normobaric hypoxia (NH), and (3) standardized exercise performed while completing a controlled breathing intervention under normoxic conditions (CB). An overview of the experimental design is presented in **Figure 1**.

**Figure 2:**
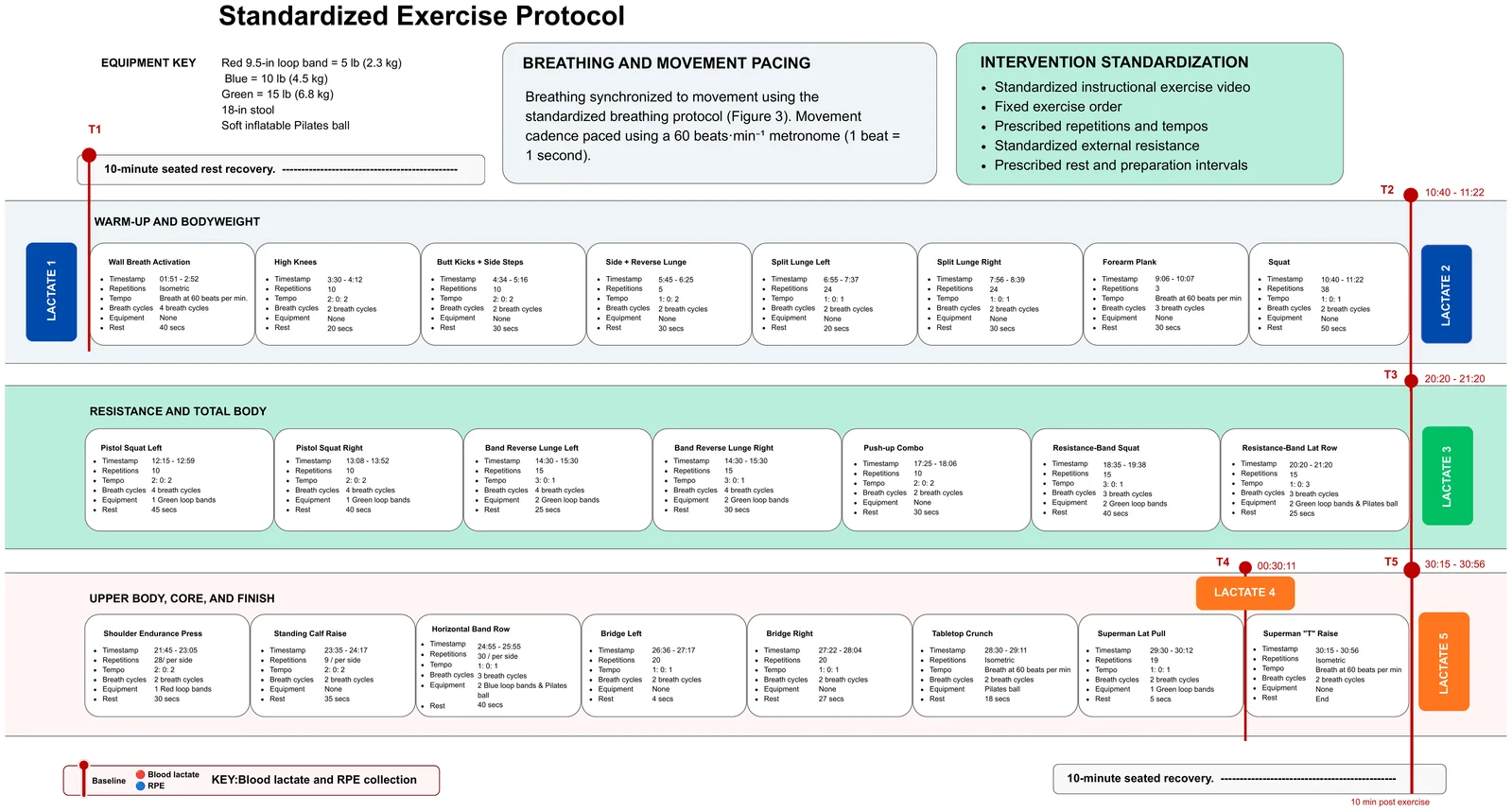
Standardized exercise protocol. Participants completed the identical 30-minute exercise protocol during each experimental condition. The protocol consisted of a standardized sequence of warm-up, resistance, upper-body, core, and postural exercises performed using prerecorded video instruction and standardized verbal cueing. Continuous pulse oximetry and heart rate monitoring were initiated before baseline measurements and maintained throughout the exercise session. Blood lactate and ratings of perceived exertion (RPE) were collected at rest (before exercise), after 10, 20, and 30 minutes of exercise, and following a 10-minute seated recovery period. Exercise cadence, movement sequence, equipment, verbal instruction, and physiological measurement timing were identical across all three experimental conditions to ensure consistent exercise workload and standardized testing procedures.

Each participant completed three laboratory visits separated by a minimum of 48 hours and no more than five days. Whenever possible, all visits were scheduled within the same two-hour period of the day to minimize potential circadian influences on physiological responses. During each visit, participants completed the identical standardized 30-minute functional exercise protocol while physiological measurements were collected according to a predetermined schedule (**Figure 2**; **Table 2**).

**Table 2:** Standardization of the experimental exercise protocol across experimental conditions.

Protocol component	CON	NH	CB
Detailed exercise prescription (exercise sequence, timestamps, repetitions, resistance, tempo, rest intervals, and physiological assessment schedule)	See **Figure 2**	See **Figure 2**	See **Figure 2**
Exercise duration	30 min	30 min	30 min
Exercise intensity	Moderate intensity	Moderate intensity	Moderate intensity
Pre-exercise safety briefing	Identical	Identical	Identical
Pre-exercise instructional video	Identical	Identical	Identical
Exercise sequence	Identical	Identical	Identical
Exercise order	Identical	Identical	Identical
Repetitions	Identical	Identical	Identical
Exercise tempo	Identical	Identical	Identical
Resistance progression	Identical	Identical	Identical
Rest and transition intervals	Identical	Identical	Identical
Standardized verbal cues	Identical	Identical	Identical
Movement cadence	Standardized using a 60 beats·min^−1^ metronome	Standardized using a 60 beats·min^−1^ metronome	Standardized using a 60 beats·min^−1^ metronome
Investigator supervision	Continuous	Continuous	Continuous
Testing environment	Identical laboratory setting	Identical laboratory setting	Identical laboratory setting
Time of testing	Same 2-hour testing window	Same 2-hour testing window	Same 2-hour testing window
Exercise equipment	Identical	Identical + hypoxic facemask	Identical
Inspired gas	Room air	15% FiO_2_	Room air
Breathing instructions	Spontaneous breathing	Spontaneous breathing	Standardized breathing cycle synchronized with movement

The exercise protocol was standardized across all three experimental conditions. The only protocol differences were the inspired oxygen concentration during the normobaric hypoxia (NH) condition and the prescribed breathing strategy during the controlled breathing (CB) condition.

The experimental conditions were completed in a fixed sequence (CON → NH → CB). This design was selected to minimize potential carryover of the learned breathing strategy into the spontaneous breathing conditions. Because the controlled breathing intervention required participants to synchronize a prescribed breathing pattern with movement execution, randomizing the breathing intervention before completion of the control and normobaric hypoxic conditions could have altered participants’ natural breathing behavior during those comparison sessions. Accordingly, the control and normobaric hypoxic conditions were completed before introducing the standardized breathing intervention.

The standardized exercise protocol incorporated identical movement sequencing, exercise duration, cadence, instructor cues, transition timing, and physiological measurement schedules across all three experimental conditions. The only intentional differences between conditions were the inspired oxygen concentration during the NH condition and the prescribed breathing strategy during the CB condition.

**Figure 3:**
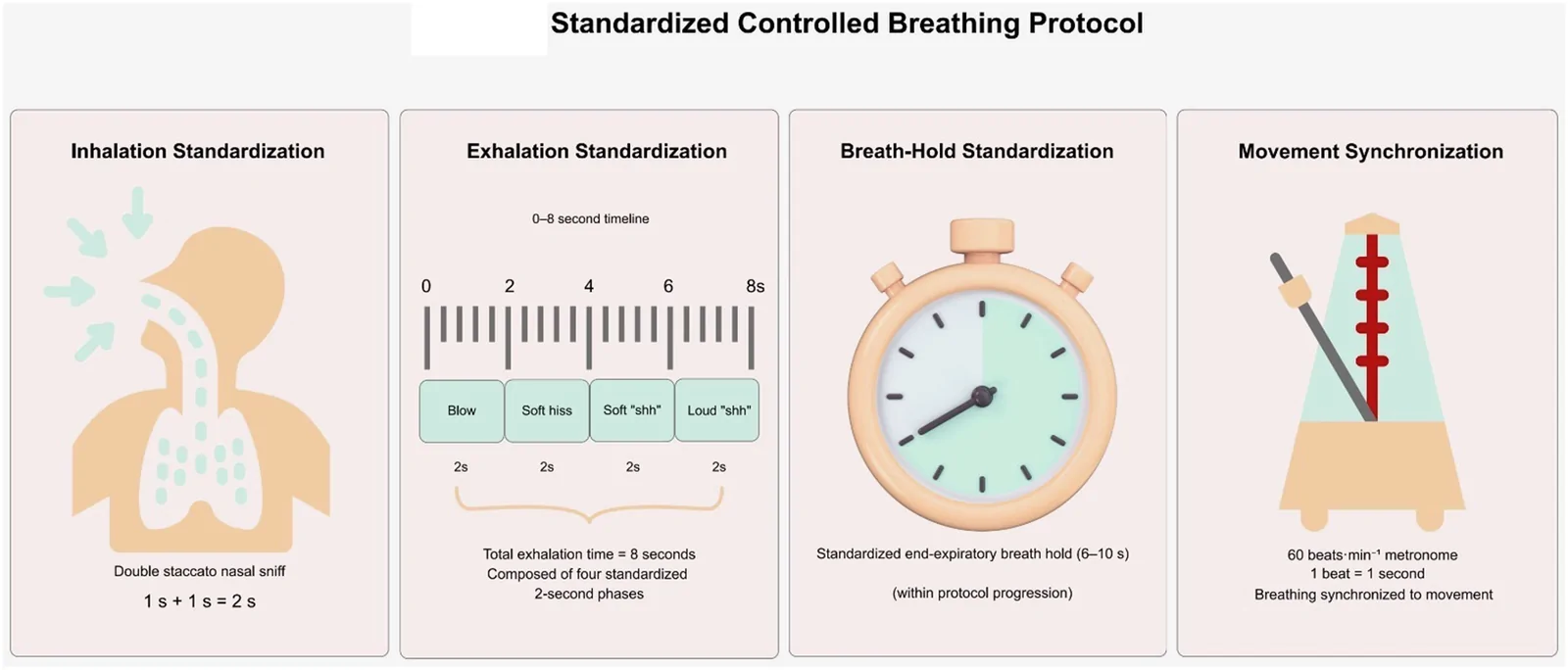
Standardization of the controlled breathing protocol. The controlled breathing intervention was standardized across all participants using a fixed breathing cadence synchronized with exercise movement. Participants performed a 2-second double staccato nasal inhalation (1 s + 1 s), followed by an 8-second exhalation consisting of four standardized 2-second phases (“blow,” “hiss,” soft “shh,” and loud “shh”), and a progressive end-expiratory breath hold ranging from 6 to 10 seconds according to the study protocol. A metronome set at 60 beats·min^−1^ (1 beat = 1 second) synchronized breathing cadence with exercise movement throughout the intervention. Standardization of inspiratory timing, expiratory structure, breath-hold duration, movement synchronization, and verbal instruction ensured a consistent breathing stimulus and improved protocol reproducibility across all participants.

Each laboratory visit followed the same sequence of events. Following baseline measurements, participants completed the standardized exercise protocol while blood lactate, peripheral oxygen saturation (SpO_2_), heart rate, and ratings of perceived exertion were collected according to the predefined measurement schedule. Blood lactate samples were obtained immediately before exercise, following 10, 20, and 30 minutes of exercise, and again following a 10-minute seated recovery period. A detailed summary of the study design, standardized exercise protocol, breathing intervention, and physiological measurement schedule is provided in **Figures 1**–**3** and **Tables 2**, **3**.

**Table 3:** Standardized controlled breathing intervention.

Component	Prescription
Inhalation	2-s staccato double nasal inhalation
Exhalation	8-s prolonged exhalation
End-expiratory breath hold	6–10-s timed breath hold following complete exhalation
Movement synchronization	Movement paused during inhalation and performed continuously throughout the exhalation and end-expiratory breath hold
Expiratory standardization	Four sequential 2-s phases: blow → hiss → soft “shh” → loud “shh”
External pacing	60 beats·min^−1^ metronome (1 beat = 1 second)

CON, control exercise; NH, normobaric hypoxia; CB, controlled breathing; FiO_2_, fraction of inspired oxygen.

Except for the inspired oxygen concentration during NH and the prescribed breathing strategy during CB, all aspects of the intervention—including participant preparation, exercise instruction, exercise sequence, duration, intensity, repetitions, movement cadence, resistance progression, rest intervals, verbal cueing, physiological assessment schedule, testing environment, and investigator supervision—were standardized across conditions. The complete exercise protocol is presented in **Figure 2**, including the exercise sequence, timestamps, prescribed repetitions, resistance, tempo, transition intervals, and blood lactate and rating of perceived exertion assessment time points.

No familiarization session was performed before experimental testing. All participants received standardized verbal instructions before each laboratory visit and completed identical exercise protocols during each experimental condition.

### 2.3 Experimental procedures and exercise protocol

Each laboratory visit followed an identical experimental sequence consisting of baseline physiological measurements, completion of the standardized exercise protocol, and post-exercise recovery measurements (**Figures 1**, **2**). All exercise sessions were conducted in the same laboratory under standardized environmental conditions using identical prerecorded instructional videos, movement sequences, exercise duration, cadence, verbal coaching, transition timing, exercise equipment, and physiological measurement schedules. The standardized exercise protocol is illustrated in **Figure 2** and summarized in **Table 2**, whereas the controlled breathing intervention is illustrated in **Figure 3** and summarized in **Table 3**.

Participants completed a standardized 30-minute functional exercise protocol designed to provide a reproducible, moderate-intensity, whole-body exercise stimulus across all three experimental conditions. The protocol consisted of a predetermined sequence of low-impact functional exercises performed using body weight, identical 10-inch (25.4 cm) latex-free mini resistance loop bands (GoFit^®^, Phoenix, AZ, USA; red, blue, and green bands providing approximately 5, 10, and 15 lb of resistance, respectively), a Franklin Method^®^ Pilates ball (Franklin Method^®^, Wetzikon, Switzerland), and a standard chair with an 18-inch (45.7 cm) seat height. The protocol incorporated standing, floor-based, upper-body, lower-body, gluteal, and core exercises. Identical exercise equipment was used for all participants throughout the study to maintain a consistent external exercise stimulus across conditions. Exercise order, exercise duration, transition periods, movement cadence, instructional cues, and physiological measurement timing were identical during every laboratory visit. As illustrated in **Figure 2**, the protocol progressed through standardized movement phases emphasizing gluteal activation, standing resistance exercises, upper-body endurance, unilateral trunk stabilization, core strengthening, and recovery. The exercise protocol was developed to provide a reproducible whole-body functional exercise stimulus while minimizing between-condition differences in external workload, thereby allowing the physiological effects of the experimental interventions to be evaluated under otherwise standardized exercise conditions.

Exercise instruction was delivered using a standardized prerecorded video narrated by the same instructor for all participants. A metronome set at 60 beats·min^−1^ was incorporated into each instructional video to maintain a consistent movement cadence throughout all three experimental conditions. Participants were instructed to synchronize their movement timing with the prerecorded instruction throughout each exercise session. Standardized verbal cues were provided to promote consistent exercise technique and intervention execution across all laboratory visits.

The intentional differences between the three experimental conditions were limited to the intervention-specific components. During the control (CON) condition, participants completed the standardized exercise protocol while breathing room air without prescribed breathing instructions. During the normobaric hypoxia (NH) condition, participants completed the identical exercise protocol while breathing normobaric hypoxic gas (FiO_2_ = 15%) delivered through a facemask connected to a commercially available hypoxic delivery system. No specific breathing pattern was prescribed during either the CON or NH conditions.

During the controlled breathing condition (**Figure 3**, **Table 3**), each breathing cycle consisted of a 2-second staccato double nasal inhalation, an 8-second standardized oral exhalation, and a 6–10-second timed end-expiratory breath hold. The inhalation consisted of two consecutive 1-second nasal inspirations. During the exhalation, participants performed the prescribed movement while maintaining continuous expiratory airflow. The exhalation was standardized using four consecutive 2-second auditory cues (“blow,” “hiss,” “soft shh,” and “loud shh”), which standardized the duration and progression of the exhalation while providing an audible method for monitoring participant adherence to the prescribed breathing pattern. At the completion of the exhalation, participants performed a timed end-expiratory breath hold while maintaining the terminal movement position before returning to the starting position during the subsequent inhalation. This breathing sequence was repeated continuously throughout the 30-minute exercise protocol. This breathing sequence was repeated continuously throughout the 30-minute exercise protocol.

To maximize intervention reproducibility, all participants received identical breathing instructions, movement demonstrations, verbal coaching, exercise progression, and physiological measurement schedules. Standardization focused on maintaining consistent movement cadence, breathing cadence, breath-hold duration, movement sequencing, exercise duration, transition timing, and physiological measurement timing, thereby minimizing variability in external workload between experimental conditions.

Blood lactate samples were collected at baseline (10 minutes before exercise), at 10, 20, and 30 minutes during exercise, and following a standardized 10-minute seated recovery period. Samples were obtained immediately after each designated exercise segment and before continuation of the exercise protocol, thereby ensuring identical sampling time points across all experimental conditions (**Figure 2**).

### 2.4 Outcome measures and instrumentation

The primary physiological outcome of this study was blood lactate concentration measured throughout the standardized exercise protocol. Additional physiological outcomes included peripheral oxygen saturation (SpO_2_), heart rate (HR), and rating of perceived exertion (RPE). All physiological measurements were collected according to the standardized assessment schedule illustrated in **Figure 2**.

#### 2.4.1 Blood lactate

Capillary blood lactate concentration was measured using the Lactate Plus™ portable blood lactate analyzer (Nova Biomedical, Waltham, MA, USA) (Tanner et al., 2010). Fingertip blood samples were obtained 10 minutes before exercise (baseline), after 10, 20, and 30 minutes of exercise, and following a standardized 10-minute seated recovery period. Exercise was briefly paused at each sampling interval to ensure identical measurement timing across all three experimental conditions (**Figure 2**). All samples were collected using standardized procedures by trained investigators in accordance with the manufacturer’s recommendations.

#### 2.4.2 Peripheral oxygen saturation

Peripheral oxygen saturation (SpO_2_) was continuously monitored using a Nonin WristOx_2_ Model 3150 pulse oximeter (Nonin Medical Inc., Plymouth, MN, USA) (Nonin Medical, Inc., 2014). The device was configured to record measurements at 4-second intervals, providing continuous estimates of arterial hemoglobin oxygen saturation by pulse oximetry throughout each experimental condition.

Throughout this manuscript, peripheral oxygen saturation (SpO_2_) refers specifically to arterial hemoglobin oxygen saturation estimated by pulse oximetry and should not be interpreted as a direct measure of skeletal muscle oxygenation, tissue oxygen availability, or cellular hypoxia, none of which were directly measured in the present study.

Continuous SpO_2_ recordings were used to characterize peripheral oxygen saturation responses throughout each experimental condition. Average low SpO_2_ during desaturation events and cumulative time spent below 88% SpO_2_ were calculated using the accompanying nVISION^®^ software (Nonin Medical Inc., Plymouth, MN, USA) (Nonin Medical, Inc., 2014).

#### 2.4.3 Heart rate

Heart rate was continuously monitored using a Polar H10 heart rate sensor (Polar Electro Oy, Kempele, Finland) paired with the Polar Beat mobile application (Polar Electro Oy, Kempele, Finland). Average heart rate during each exercise session was calculated for statistical analyses. The Polar H10 has demonstrated excellent agreement with electrocardiography during exercise and was selected to provide accurate continuous heart rate monitoring (Gilgen-Ammann et al., 2019).

#### 2.4.4 Rating of perceived exertion

Perceived exertion was assessed using the Borg 6–20 Rating of Perceived Exertion (RPE) scale (Borg, 1982). Participants reported RPE 10 minutes before exercise (baseline), after 10, 20, and 30 minutes of exercise, and following the 10-minute seated recovery period according to the standardized assessment schedule illustrated in **Figure 2**.

All physiological measurements were collected at identical time points across the three experimental conditions to ensure standardized data collection and facilitate direct comparisons between interventions.

### 2.5 Statistical analysis

Statistical analyses were performed using R version 4.5.2 (R Foundation for Statistical Computing, Vienna, Austria) with the lme4 (version 1.1-37), lmerTest (version 3.1-3), and emmeans (version 2.0.0) packages. Linear mixed-effects models were used to account for the repeated-measures crossover design. Denominator degrees of freedom were estimated using the Kenward–Roger approximation, and Type III analyses of variance (ANOVA) were used to evaluate fixed effects. When significant omnibus effects were identified, Tukey-adjusted pairwise comparisons of estimated marginal means were performed. Statistical significance was accepted at p < 0.05.

Repeated-measures outcomes, including blood lactate concentration and rating of perceived exertion (RPE), were analyzed using linear mixed-effects models with fixed effects for condition, time, and the condition × time interaction. Random intercepts were included for participant and participant-by-condition. Single-session summary outcomes, including maximum blood lactate, blood lactate area under the curve (AUC), baseline-adjusted blood lactate AUC, average heart rate, average low peripheral oxygen saturation (SpO_2_), and cumulative time spent below 88% SpO_2_, were analyzed using linear mixed-effects models with condition as a fixed effect and participant included as a random intercept.

Blood lactate AUC was calculated using the trapezoidal rule across the five sampling time points (baseline, 10, 20, and 30 minutes of exercise, and 10 minutes post-exercise). Baseline-adjusted AUC was calculated by subtracting each participant’s baseline blood lactate concentration from all subsequent measurements before AUC calculation. Average heart rate was calculated as the mean heart rate recorded throughout the 30-minute exercise session. Average low SpO_2_ represents the software-derived average peripheral oxygen saturation during identified desaturation events, as reported by the Nonin nVISION^®^ software, and should not be interpreted as the average SpO_2_ across the entire exercise session.

Model assumptions were evaluated by visual inspection of residual plots and assessment of residual normality. Effect sizes for omnibus tests are reported as partial η^2^. Data are presented as estimated marginal means ± standard error (SE), unless otherwise indicated.

## 3 Results

### 3.1 Blood lactate

Blood lactate was evaluated using four complementary analyses to characterize the metabolic response to each intervention: repeated-measures blood lactate concentrations throughout exercise and recovery, maximum blood lactate concentration, blood lactate area under the curve (AUC), and baseline-adjusted blood lactate AUC.

#### 3.1.1 Blood lactate during exercise

**Figure 4:**
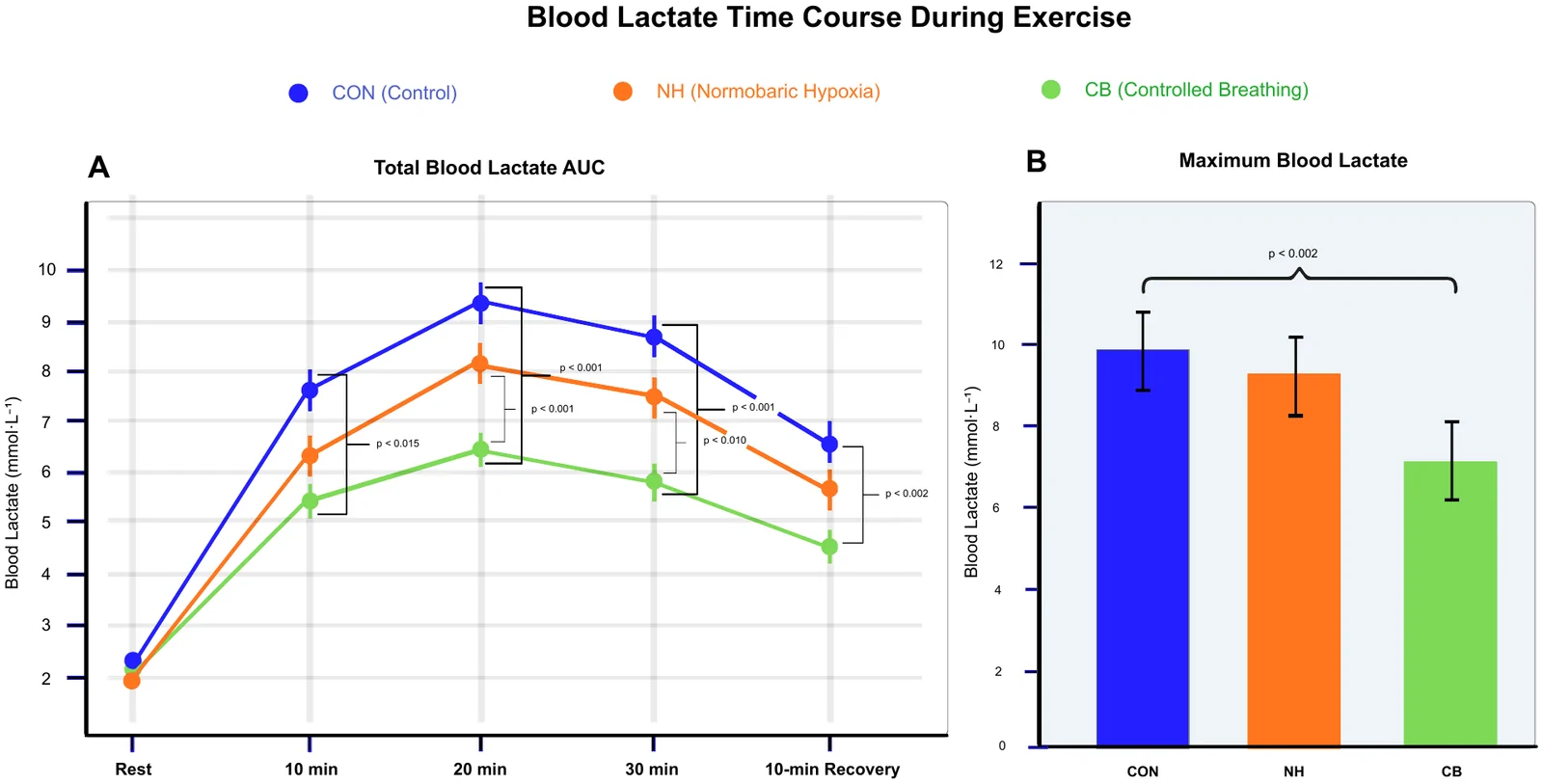
Blood lactate time course during exercise. Mean ± SEM blood lactate concentrations measured at rest, 10, 20, and 30 minutes during exercise, and following a 10-minute seated recovery period under the control (CON), normobaric hypoxia (NH), and controlled breathing (CB) conditions. Linear mixed-effects modeling demonstrated significant main effects of condition (p < 0.001) and time (p < 0.001), as well as a significant condition × time interaction (p = 0.007). Tukey-adjusted pairwise comparisons are shown. During exercise and recovery, controlled breathing elicited significantly lower blood lactate concentrations compared with control at 10, 20, and 30 minutes and recovery, and compared with normobaric hypoxia at 20 and 30 minutes.

Repeated-measures analysis demonstrated a significant main effect of condition (F(2,28) = 18.001, *p* < 0.001, partial η^2^ = 0.563), a significant main effect of time (F(4,168) = 96.339, *p* < 0.001, partial η^2^ = 0.696), and a significant condition × time interaction (F(8,168) = 2.728, *p* = 0.007, partial η^2^ = 0.115) (**Table 4**; **Figure 4**).

**Table 4:** Summary of linear mixed-effects model analyses for primary and additional physiological outcomes.

Outcome	Effect	F (df)	p	Partial η^2^	Significant pairwise comparisons
Blood Lactate	Condition	F(2,28)=18.001	<0.001	0.563	CON > CB (10, 20, 30 min, recovery); NH > CB (20, 30 min)
	Time	F(4,168)=96.339	<0.001	0.696	—
	Condition × Time	F(8,168)=2.728	0.007	0.115	—
Maximum Blood Lactate	Condition	F(2,28)=7.506	0.002	0.349	CON > CB
Blood Lactate AUC	Condition	F(2,28)=17.720	<0.001	0.559	CON > NH; CON > CB; NH > CB
Average Heart Rate	Condition	F(2,28)=10.986	<0.001	0.440	CON > CB; NH > CB
Average Peripheral Oxygen Saturation	Condition	F(2,28)=31.184	<0.001	0.690	CON > NH; CON > CB; CB > NH
Rating of Perceived Exertion	Condition	F(2,28)=1.026	0.371	0.068	None
	Time	F(4,168)=307.458	<0.001	0.880	—
	Condition × Time	F(8,168)=0.922	0.500	0.042	—

Values represent Type III linear mixed-effects models with Kenward–Roger degrees of freedom. Only statistically significant pairwise comparisons are reported. Tukey-adjusted *post hoc* comparisons are presented as reported in the final statistical analysis.

As illustrated in **Figure 4**, blood lactate increased progressively throughout exercise in all three conditions. However, lactate accumulation was consistently attenuated during controlled breathing. Tukey-adjusted pairwise comparisons demonstrated significantly lower blood lactate concentrations during controlled breathing than during the control condition at 10 minutes (*p* = 0.015), 20 minutes (*p* < 0.001), 30 minutes (*p* < 0.001), and 10 minutes post-exercise (*p* = 0.002). Blood lactate concentrations were also significantly lower during controlled breathing than during normobaric hypoxia at 20 minutes (*p* = 0.001) and 30 minutes (*p* = 0.010). No significant differences were observed between the control and normobaric hypoxia conditions at any time point.

#### 3.1.2 Maximum blood lactate

The attenuation in blood lactate accumulation was reflected in the peak exercise response (**Figure 4**). Maximum blood lactate differed significantly among conditions (F(2,28) = 7.506, *p* = 0.002, partial η^2^ = 0.349) (**Table 4**). Maximum blood lactate was significantly lower during controlled breathing than during the control condition (*p* = 0.002), whereas no significant differences were observed between the control and normobaric hypoxia conditions (*p* = 0.334) or between normobaric hypoxia and controlled breathing (*p* = 0.060).

#### 3.1.3 Blood lactate area under the curve

**Figure 5:**
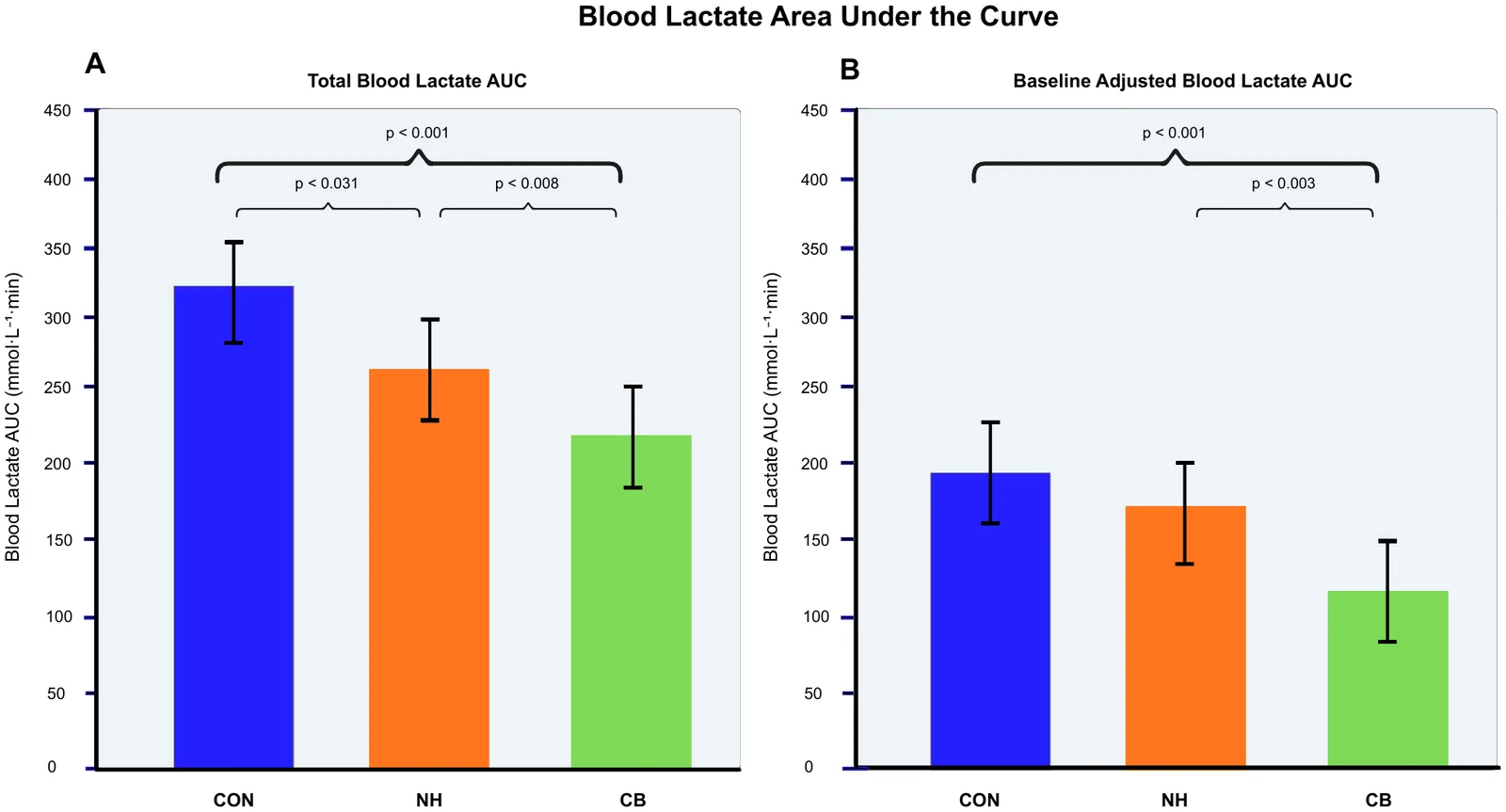
Blood lactate area under the curve. Area under the blood lactate concentration–time curve (AUC) calculated from rest through the 10-minute seated recovery period under the control (CON), normobaric hypoxia (NH), and controlled breathing (CB) conditions. Values are mean ± SEM (mmol·L^−1^·min). Linear mixed-effects modeling demonstrated a significant main effect of condition (p < 0.001). Tukey-adjusted pairwise comparisons are shown. Controlled breathing resulted in a significantly lower lactate AUC compared with control and normobaric hypoxia, and normobaric hypoxia was significantly lower than control.

Consistent with the repeated-measures findings, cumulative blood lactate exposure also differed significantly among conditions (F(2,28) = 17.720, *p* < 0.001, partial η^2^ = 0.559) (**Table 4**; **Figure 5**). As illustrated in **Figure 5**, controlled breathing produced the lowest blood lactate AUC across the exercise session. Blood lactate AUC was significantly lower during controlled breathing than during both the control (*p* < 0.001) and normobaric hypoxia (*p* = 0.008) conditions. Blood lactate AUC was also significantly lower during normobaric hypoxia than during the control condition (*p* = 0.031).

#### 3.1.4 Baseline-adjusted blood lactate area under the curve

After adjustment for baseline blood lactate concentration, significant differences between conditions remained (F(2,28) = 12.694, *p* < 0.001, partial η^2^ = 0.476) (**Table 4**). Baseline-adjusted blood lactate AUC was significantly lower during controlled breathing than during both the control (*p* < 0.001) and normobaric hypoxia (*p* = 0.003) conditions, whereas no significant difference was observed between the control and normobaric hypoxia conditions (*p* = 0.450).

Collectively, these complementary analyses demonstrated a consistent reduction in the blood lactate response during controlled breathing. Compared with the control condition, controlled breathing reduced blood lactate accumulation throughout exercise, lowered peak blood lactate concentration, decreased cumulative blood lactate exposure, and maintained these differences after adjustment for baseline values. In most analyses, controlled breathing also produced a lower blood lactate response than normobaric hypoxia.

### 3.2 Average heart rate

**Figure 6:**
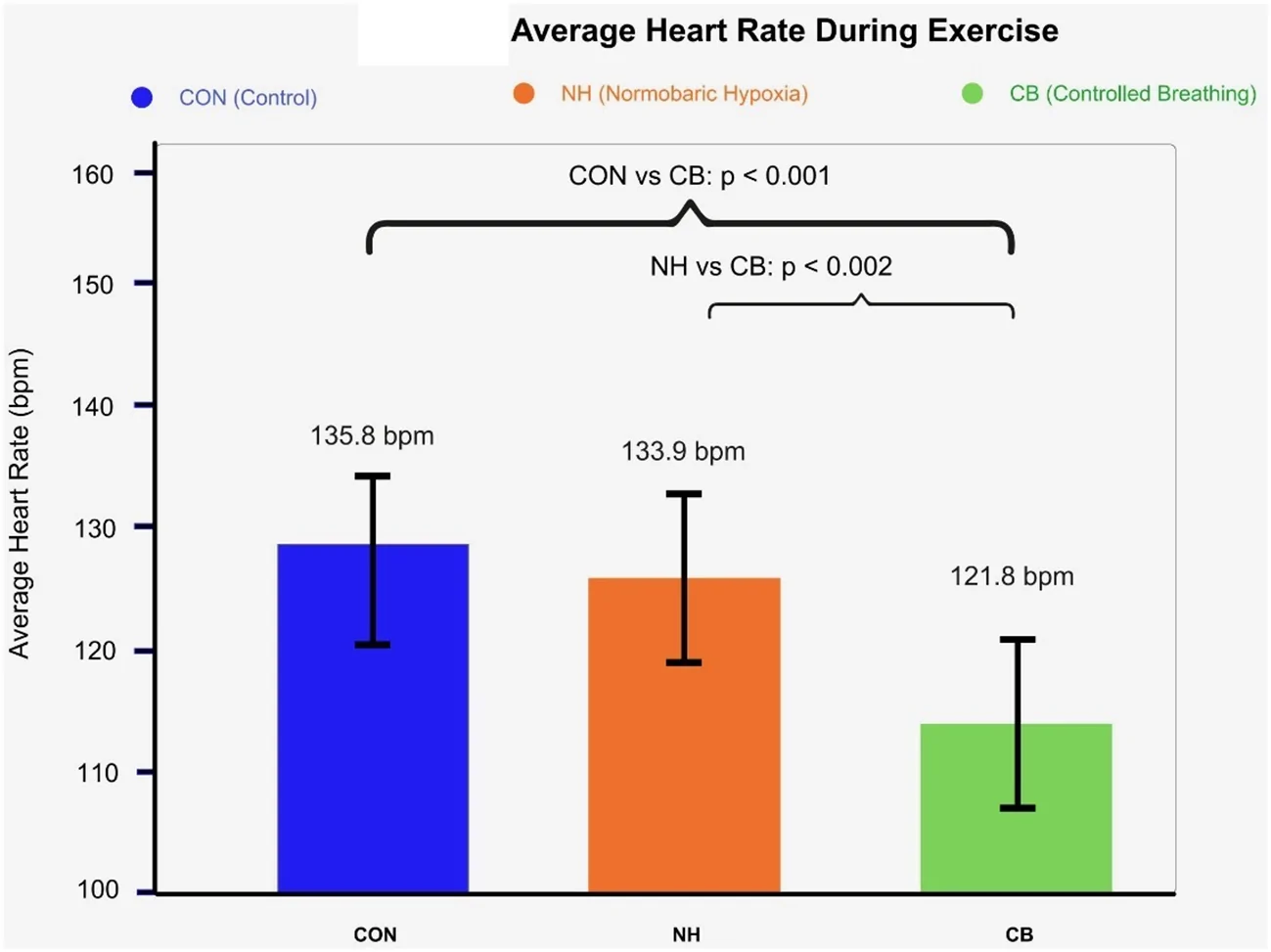
Average heart rate during exercise. Estimated marginal mean heart rate (± SE) measured during the standardized 30-minute exercise protocol under the control (CON), normobaric hypoxia (NH), and controlled breathing (CB) conditions (n = 15). Average heart rate differed significantly among conditions (F(2,28) = 10.99, p < 0.001). Tukey-adjusted pairwise comparisons demonstrated lower average heart rate during CB compared with CON (p < 0.001) and NH (p = 0.002), whereas CON and NH did not differ significantly (p = 0.834). Error bars represent ±1 SE. CON, control; NH, normobaric hypoxia; CB, controlled breathing.

Average heart rate differed significantly among conditions (F(2,28) = 10.986, p < 0.001, partial η^2^ = 0.440) (**Table 4**; **Figure 6**). Estimated marginal mean heart rates were 135.8 ± 3.0 bpm for the control condition, 133.9 ± 3.0 bpm for normobaric hypoxia, and 121.8 ± 3.0 bpm for controlled breathing.

Average heart rate was significantly lower during controlled breathing than during both the control (p < 0.001) and normobaric hypoxia (p = 0.002) conditions. No significant difference was observed between the control and normobaric hypoxia conditions (p = 0.834). As illustrated in **Figure 6**, controlled breathing elicited the lowest average heart rate across the three experimental conditions, consistent with the reduced blood lactate response observed during exercise.

### 3.3 Peripheral oxygen saturation

**Figure 7:**
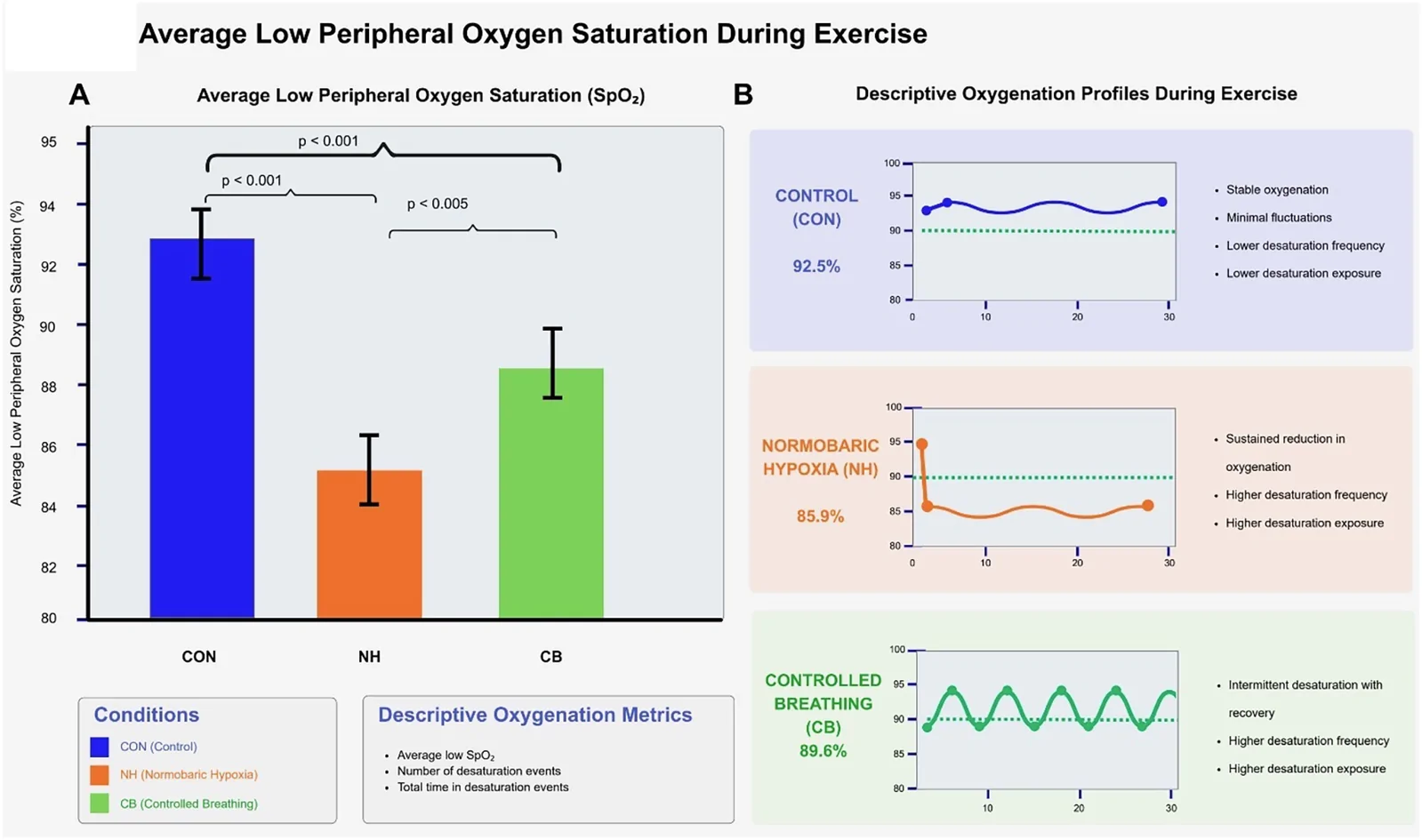
Peripheral oxygen saturation during exercise: group comparisons and representative oxygenation patterns. **(A)** Average low peripheral oxygen saturation (SpO_2_) derived from continuous pulse oximetry recordings throughout the 30-minute exercise bout under the control (CON), normobaric hypoxia (NH), and controlled breathing (CB) conditions (n = 15). Statistical comparisons were conducted using linear mixed-effects models. Tukey-adjusted pairwise comparisons demonstrated lower average low SpO_2_during normobaric hypoxia compared with control (p = 0.0074) and controlled breathing (p = 0.0072), and lower average low SpO_2_ during controlled breathing compared with control (p = 0.0055). **(B)** Descriptive oxygenation profiles illustrating the characteristic pattern of peripheral oxygen saturation during exercise under each experimental condition. Control was characterized by relatively stable oxygenation with minimal fluctuations and lower desaturation frequency and exposure; normobaric hypoxia was characterized by a sustained reduction in oxygenation with higher desaturation frequency and exposure; and controlled breathing was characterized by intermittent reductions in oxygen saturation followed by recovery, with higher desaturation frequency and exposure. The profiles in panel B are descriptive representations of the observed oxygenation patterns and are not inferential statistical comparisons.

Average low peripheral oxygen saturation differed significantly among conditions (F(2,28) = 31.184, p < 0.001, partial η^2^ = 0.690) (**Table 4**; **Figure 7**). Estimated marginal means were 92.45 ± 0.67% for the control condition, 85.91 ± 0.67% for normobaric hypoxia, and 89.60 ± 0.67% for controlled breathing. Average low SpO_2_ was significantly lower during normobaric hypoxia than during both the control (p < 0.001) and controlled breathing (p < 0.001) conditions. Controlled breathing also demonstrated significantly lower average low SpO_2_ than the control condition (p = 0.005).

To further characterize the oxygenation response, cumulative time spent below 88% SpO_2_ also differed significantly among conditions (F(2,28) = 32.160, p < 0.001, partial η^2^ = 0.697) (**Table 4**). Estimated marginal means were 1.41 ± 1.14 min for the control condition, 12.83 ± 1.14 min for normobaric hypoxia, and 2.43 ± 1.14 min for controlled breathing. Normobaric hypoxia resulted in significantly greater cumulative time below 88% SpO_2_ than both the control (p < 0.001) and controlled breathing (p < 0.001) conditions. No significant difference was observed between the control and controlled breathing conditions (p = 0.793).

As illustrated in **Figure 7**, the three interventions produced distinct oxygenation profiles. The control condition maintained relatively stable peripheral oxygen saturation throughout exercise with minimal desaturation. Normobaric hypoxia produced a sustained reduction in peripheral oxygen saturation accompanied by the greatest cumulative time below 88% SpO_2_. In contrast, controlled breathing produced repeated intermittent desaturation episodes followed by recovery between events, resulting in significantly lower average low SpO_2_ than the control condition while producing substantially less cumulative time below 88% than normobaric hypoxia.

### 3.4 Rating of perceived exertion

**Figure 8:**
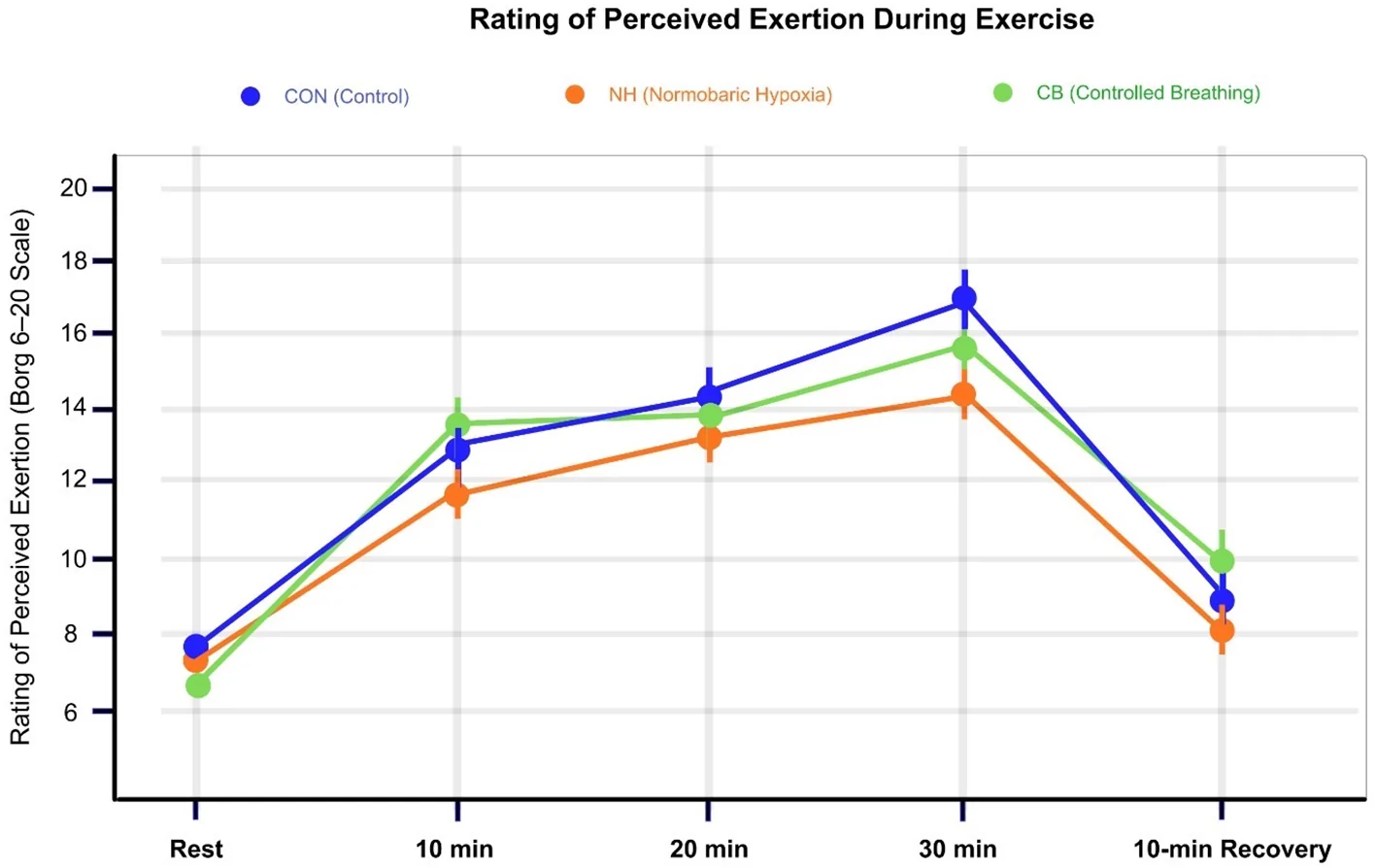
Rating of perceived exertion during exercise. Estimated marginal means (± SE) for rating of perceived exertion (RPE; Borg 6–20 scale) measured at rest (before equipment), after 10, 20, and 30 minutes of exercise, and following a 10-minute seated recovery period during the control (CON), normobaric hypoxia (NH), and controlled breathing (CB) conditions (n = 15). Linear mixed-effects analysis demonstrated a significant main effect of time [F(4,168) = 307.458, p < 0.001], indicating that perceived exertion increased throughout exercise and declined during recovery. There was no significant main effect of condition [F(2,28) = 1.026, p = 0.371] and no condition × time interaction [F(8,168) = 0.922, p = 0.500], indicating that perceived exertion changed similarly across all three experimental conditions. Error bars represent ±1 SE. CON, control; NH, normobaric hypoxia; CB, controlled breathing; RPE, rating of perceived exertion.

Rating of perceived exertion did not differ significantly among conditions (F(2,28) = 1.026, p = 0.371, partial η^2^ = 0.068), and no significant condition × time interaction was observed (F(8,168) = 0.922, p = 0.500, partial η^2^ = 0.042). However, RPE increased significantly over time (F(4,168) = 307.458, p < 0.001, partial η^2^ = 0.880) (**Table 4**; **Figure 8**). Estimated marginal mean RPE values were 10.69 ± 0.28 for the control condition, 10.39 ± 0.28 for normobaric hypoxia, and 10.71 ± 0.28 for controlled breathing. No significant pairwise differences were observed between conditions (all p ≥ 0.426).

As illustrated in **Figure 8**, perceived exertion increased progressively throughout exercise in all three conditions, but the pattern of increase was nearly identical between interventions. Despite significant differences in blood lactate accumulation, average heart rate, and peripheral oxygen saturation, participants perceived a similar level of exercise intensity across all three experimental conditions.

## 4 Discussion

### 4.1 Principal findings

The primary finding of this exploratory crossover study was that the standardized controlled breathing intervention was associated with significantly lower blood lactate concentrations during moderate-intensity exercise than both the normobaric hypoxic and control conditions despite producing reductions in peripheral oxygen saturation during exercise. Participants also demonstrated a lower average heart rate during the controlled breathing intervention, whereas ratings of perceived exertion remained similar across all three experimental conditions (**Figures 4**-**8**; **Table 4**). Together, these findings indicate that interventions capable of reducing peripheral oxygen saturation do not necessarily produce similar metabolic responses during exercise and suggest that physiological factors beyond oxygen availability alone, including differences in ventilatory physiology, respiratory muscle energy demand, autonomic regulation, and breathing–movement coordination, may contribute to metabolic regulation during exercise (Dempsey et al., 2006; Paced breathing in roller-ski skating: Effects on metabolic rate and poling forces, 2007; Brooks, 2018).

These findings were contrary to our original hypothesis. Previous investigations have shown that both hypoxic exercise and breathing interventions capable of reducing peripheral oxygen saturation are commonly associated with increased blood lactate accumulation (Woorons et al., 2010; Woorons et al., 2019; Woorons et al., 2020). Consistent with these findings and our own preliminary data (Brindisi et al., 2024), we anticipated that both the controlled breathing and normobaric hypoxic interventions would produce greater blood lactate concentrations than the control condition. Instead, the controlled breathing intervention consistently demonstrated lower blood lactate concentrations throughout exercise and recovery than both comparison conditions. This pattern was evident across repeated blood lactate measurements, peak blood lactate concentration, blood lactate area under the curve (AUC), and baseline-adjusted AUC, demonstrating a sustained reduction in the cumulative metabolic response throughout the exercise protocol rather than isolated differences at individual sampling time points.

The present study was designed as a direct extension of our previous pilot investigation, which demonstrated that the standardized controlled breathing intervention used here produced intermittent reductions in peripheral oxygen saturation during moderate-intensity exercise comparable to those achieved with intermittent hypoxic training (Brindisi et al., 2024). Accordingly, the objective of the present study was not to determine whether the breathing intervention could reduce peripheral oxygen saturation, but rather to determine whether a breathing strategy capable of inducing peripheral oxygen desaturation would elicit metabolic responses similar to those observed during normobaric hypoxic exercise. Contrary to our original hypothesis, although the controlled breathing intervention successfully reduced peripheral oxygen saturation, it was associated with significantly lower blood lactate concentrations than both the control and normobaric hypoxia conditions. This unexpected dissociation between peripheral oxygen saturation and blood lactate accumulation represents a principal novel finding of the present study.

The physiological mechanisms responsible for these unexpected findings cannot be determined from the present study because this investigation was designed to compare physiological responses between interventions rather than identify underlying mechanisms. Accordingly, the present findings should be interpreted as generating hypotheses for future investigation rather than establishing causal relationships. The combination of lower blood lactate concentrations, lower average heart rate, unchanged ratings of perceived exertion, and reduced peripheral oxygen saturation during the controlled breathing intervention suggests a more complex relationship between oxygenation and metabolic regulation than would be predicted by pulse oximetry alone.

### 4.2 Comparison with previous literature

The present findings differ from those reported in many previous investigations examining exercise performed under hypoxic conditions and voluntary hypoventilation. Studies of exercise performed under reduced inspired oxygen availability have consistently demonstrated greater blood lactate accumulation than equivalent exercise performed under normoxic conditions despite similar external workloads, supporting the concept that hypoxia increases reliance on glycolytic metabolism during exercise (Woorons et al., 2010; Faiss et al., 2013; Girard et al., 2017; Woorons et al., 2019; Woorons et al., 2020). Likewise, Woorons and colleagues demonstrated that voluntary hypoventilation protocols capable of producing transient reductions in arterial oxygen saturation also resulted in greater blood lactate accumulation, suggesting that restricting ventilation relative to metabolic demand augments glycolytic stress during exercise (Woorons et al., 2010; Woorons et al., 2019; Woorons et al., 2020). Collectively, these investigations provided the physiological rationale for our original hypothesis that both the normobaric hypoxic and controlled breathing interventions would increase blood lactate accumulation relative to the control condition.

The present findings, however, differed from these expectations. Although previous investigations have generally reported greater blood lactate accumulation during hypoxic exercise than during equivalent normoxic exercise (Faiss et al., 2013; Girard et al., 2017; Schiffer et al., 2017; Faulhaber et al., 2020), the normobaric hypoxic intervention in the present study, while successfully reducing peripheral oxygen saturation, did not produce significantly higher blood lactate concentrations than the control condition. More notably, the standardized controlled breathing intervention produced significantly lower blood lactate concentrations throughout exercise and recovery than both the control and normobaric hypoxic conditions despite also producing reductions in peripheral oxygen saturation during exercise. Together, these findings suggest that interventions capable of producing comparable reductions in peripheral oxygen saturation do not necessarily elicit similar metabolic responses and that oxygen availability measured by pulse oximetry alone may not fully explain blood lactate regulation during moderate-intensity exercise.

Several important methodological differences should be considered when comparing the present findings with previous investigations of voluntary hypoventilation. Unlike the protocols developed by Woorons and colleagues, the breathing intervention employed in the present study was not designed as a traditional voluntary hypoventilation protocol (Woorons et al., 2010; Woorons et al., 2019; Woorons et al., 2020). Instead, participants performed a standardized breathing strategy incorporating a staccato double nasal inhalation, prolonged timed exhalation, end-expiratory breath holds, synchronized breathing and movement, and periods of recovery breathing throughout the exercise session. These components were selected to maximize intervention standardization and reproducibility rather than to isolate the physiological effects of any single breathing maneuver. Consequently, the present intervention should be considered a distinct breathing strategy rather than a direct replication of previously published voluntary hypoventilation paradigms.

The present study also extends our previous pilot investigation, which demonstrated that this standardized breathing intervention produced intermittent reductions in peripheral oxygen saturation comparable to those achieved during intermittent hypoxic exercise but did not assess metabolic responses (Brindisi et al., 2024). The objective of the present study was therefore to determine whether a breathing strategy capable of inducing peripheral oxygen desaturation would produce metabolic responses similar to those observed during normobaric hypoxia. Contrary to our original hypothesis, the present findings demonstrate that similar reductions in peripheral oxygen saturation were associated with markedly different blood lactate responses depending on the intervention employed.

The present findings may also be interpreted in the context of previous investigations examining paced breathing and breathing–movement synchronization during exercise. Fabre and colleagues reported that paced breathing and synchronized respiration can modify physiological and perceptual responses during dynamic exercise independently of whole-body oxygen consumption, suggesting that breathing pattern itself represents an important physiological variable rather than simply a consequence of metabolic demand (Fabre et al., 2007; Paced breathing in roller-ski skating: Effects on metabolic rate and poling forces, 2007). Although the breathing intervention employed in the present study differed substantially from those protocols, these observations support the concept that different breathing strategies may produce distinct physiological responses despite similar external workloads and comparable reductions in peripheral oxygen saturation.

Accordingly, direct comparisons between the present study and previous investigations of hypoxic exercise or voluntary hypoventilation should be interpreted cautiously. Rather than contradicting earlier work, the present findings suggest that breathing interventions capable of producing reductions in peripheral oxygen saturation may represent physiologically distinct stimuli depending on the breathing pattern employed, the duration and timing of breath holds, breathing–movement synchronization, the exercise protocol, and the overall structure of the intervention. Future mechanistic studies directly comparing standardized breathing interventions, voluntary hypoventilation, and normobaric hypoxia under identical experimental conditions will be necessary to determine which physiological characteristics are responsible for the divergent metabolic responses observed in the present study.

### 4.3 Potential physiological explanations

The physiological mechanisms underlying the lower blood lactate response observed during the controlled breathing intervention cannot be determined from the present study. Because this investigation was designed to compare physiological responses between interventions rather than identify underlying mechanisms, the present findings should be interpreted as generating hypotheses for future investigation rather than establishing causal relationships. Nevertheless, several physiological mechanisms warrant consideration and may help explain the observed dissociation between reductions in peripheral oxygen saturation measured by pulse oximetry and blood lactate accumulation.

One potential explanation relates to the metabolic cost of ventilation. The respiratory muscles require substantial oxygen and ATP during exercise, and increases in ventilatory work can contribute meaningfully to whole-body energy expenditure, particularly as exercise intensity increases (Harms et al., 1997; Harms et al., 1998; Dempsey et al., 2006). Consequently, alterations in breathing pattern have the potential to influence overall physiological strain independent of external workload (Dempsey et al., 2006). The standardized breathing strategy employed during the controlled breathing intervention may have altered the metabolic cost of ventilation and respiratory muscle energy demand, thereby attenuating activation of the respiratory muscle metaboreflex, reducing sympathetic activation, and contributing to the lower average heart rate and blood lactate concentrations observed during exercise (Harms et al., 1998; Sheel, 2002; Dempsey et al., 2006). Although ventilatory work, respiratory muscle activity, and respiratory gas exchange were not measured directly, this mechanism represents one plausible explanation that warrants further investigation.

A second potential mechanism involves alterations in carbon dioxide physiology. The standardized breathing protocol incorporated prolonged exhalation, timed end-expiratory breath holds, and recovery breathing throughout exercise, each of which may have influenced carbon dioxide handling and acid-base regulation differently than spontaneous breathing or exercise performed under normobaric hypoxia (Woorons et al., 2010; Woorons et al., 2019; Woorons et al., 2020). Carbon dioxide availability has been proposed to influence lactate kinetics during exercise, although the direction and magnitude of these effects appear to depend on the specific physiological context (McLellan, 1991; Woorons et al., 2010). Because end-tidal carbon dioxide, arterial blood gases, and respiratory gas exchange were not assessed, the contribution of altered carbon dioxide physiology cannot be determined and therefore remains speculative. Future investigations incorporating these measurements will be necessary to clarify the role of carbon dioxide physiology in the metabolic responses observed during controlled breathing exercise.

Another distinguishing feature of the present intervention was the intentional synchronization of respiration with movement throughout the exercise protocol. Although this differs from traditional locomotor respiratory coupling, in which breathing becomes naturally entrained to rhythmic locomotion, previous investigations have demonstrated that coordinated breathing and movement are influenced predominantly by neuromechanical interactions rather than metabolic stimuli alone (Fabre et al., 2007). Complementary work by Fabre and colleagues further demonstrated that paced breathing during dynamic exercise can alter physiological and perceptual responses independently of whole-body oxygen consumption, supporting the concept that breathing pattern itself represents an important physiological variable during exercise (Paced breathing in roller-ski skating: Effects on metabolic rate and poling forces, 2007). Unlike spontaneous breathing, participants in the present study synchronized every exercise repetition with a standardized breathing cadence incorporating prolonged exhalation and timed end-expiratory breath holds. Although the breathing–movement synchronization protocol was rigorously standardized across participants, the present study did not directly measure respiratory mechanics, ventilatory responses, respiratory muscle activation, or neural control of breathing. Consequently, the mechanisms underlying the observed reductions in blood lactate remain speculative. Future investigations incorporating direct assessments of respiratory mechanics, respiratory muscle function, neural drive, carbon dioxide kinetics, and gas exchange are warranted.

An additional consideration is that blood lactate concentration reflects the dynamic balance between lactate production and lactate clearance rather than the rate of lactate production alone (Brooks, 1985; Gladden, 2004; Brooks, 2018; Brooks, 2020). Consequently, the lower blood lactate concentrations observed during the controlled breathing intervention could theoretically reflect reduced lactate production, enhanced lactate clearance, or a combination of both processes. Because blood lactate concentration was the primary metabolic variable assessed, the present study cannot distinguish among these possibilities. This distinction is particularly relevant given the contemporary understanding of lactate as a continuously produced and utilized metabolic intermediate that serves as an important fuel source and signaling molecule rather than simply a marker of anaerobic metabolism (Brooks, 1985; Gladden, 2004; Brooks, 2018; Brooks, 2020).

Beyond these potential mechanisms, blood lactate accumulation is regulated by numerous interacting physiological systems, including substrate utilization, sympathetic activation, hormonal responses, ventilatory regulation, and mitochondrial oxidative capacity (Brooks, 1985; Gladden, 2004; Mazzeo, 2008; Brooks, 2018; Brooks, 2020). The lower average heart rate observed during the controlled breathing intervention may suggest altered physiological regulation during exercise. Previous studies have reported that slow, controlled breathing, particularly strategies incorporating prolonged exhalation, may enhance parasympathetic modulation and reduce physiological stress, which could theoretically attenuate catecholamine-mediated stimulation of glycolysis and contribute to lower blood lactate concentrations (Mazzeo, 2008; Ferrari et al., 2011; Perrey and Ferrari, 2018). However, autonomic activity, catecholamine concentrations, substrate metabolism, ventilatory efficiency, and other regulatory variables were not measured in the present study. Consequently, the relative contribution of these physiological processes cannot be determined from the present findings.

Finally, the present findings highlight the limitations of interpreting peripheral oxygen saturation measured by pulse oximetry as a surrogate for the complex physiological processes governing metabolic regulation during exercise (Jubran, 2015). Pulse oximetry provides an estimate of arterial oxygen saturation but does not directly assess skeletal muscle oxygenation, tissue oxygen extraction, local oxygen utilization, regional blood flow, or the temporal fluctuations in oxygen availability that may occur during intermittent breath-hold exercise (Ferrari et al., 2011; Perrey and Ferrari, 2018). Accordingly, similar reductions in peripheral oxygen saturation should not be interpreted as evidence of equivalent tissue oxygenation or metabolic conditions throughout exercise (Perrey and Ferrari, 2018). Future investigations incorporating near-infrared spectroscopy, indirect calorimetry, respiratory gas analysis, arterial blood gases, respiratory muscle electromyography, respiratory muscle oxygenation, end-tidal carbon dioxide monitoring, and additional measures of autonomic and metabolic function will be essential for determining the physiological mechanisms responsible for the lower blood lactate response observed during the controlled breathing intervention.

### 4.4 Perceived exertion

An additional noteworthy finding was that ratings of perceived exertion (RPE) remained similar across all three experimental conditions despite significant differences in blood lactate concentration and average heart rate. This observation is particularly interesting because a meta-analysis by Chen et al. (2002) reported a moderate positive association (r = 0.57) between blood lactate concentration and ratings of perceived exertion, supporting the expectation that lower blood lactate concentrations would generally be accompanied by lower perceived exertion (Chen et al., 2002). Likewise, Noble et al. (1983), Green et al. (2005), and Irving et al. (2006) demonstrated consistent relationships among blood lactate concentration, heart rate, and perceived exertion during incremental and steady-state exercise (Noble et al., 1983; Green et al., 2005; Irving et al., 2006). Based on these previous findings, one might reasonably have expected the lower blood lactate concentrations and average heart rate observed during the controlled breathing intervention to be accompanied by lower perceived exertion. However, this was not observed, suggesting that subjective perceptions of exercise intensity do not necessarily parallel individual physiological variables during moderate-intensity exercise. Rather, perceived exertion is widely recognized as a multidimensional construct that integrates afferent feedback from the cardiovascular, respiratory, metabolic, neuromuscular, and central nervous systems, together with cognitive and psychological influences, rather than reflecting any single physiological response (Borg, 1982; Noble and Robertson, 1996; Marcora, 2009; Pageaux, 2016).

Several explanations may account for the preservation of perceived exertion observed in the present study. Although the standardized controlled breathing intervention was associated with lower blood lactate concentrations and average heart rate and may have altered the metabolic cost of ventilation, participants were simultaneously required to consciously regulate a prescribed breathing pattern throughout the exercise session. The cognitive and attentional demands associated with maintaining synchronized breathing and movement may therefore have offset any reduction in physiological strain (Noble and Robertson, 1996; Marcora, 2009; Pageaux, 2016). In addition, participants in the normobaric hypoxic condition exercised while wearing a hypoxic delivery mask, which may have influenced breathing comfort and respiratory perception independently of metabolic demand. More broadly, because perceived exertion reflects the integration of numerous physiological and psychological inputs, changes in individual physiological variables do not necessarily produce corresponding changes in the overall perception of effort (Marcora, 2009; Pageaux, 2016). Because respiratory effort, ventilatory parameters, dyspnea, respiratory muscle activity, affective responses, and cognitive workload were not directly measured, these explanations remain speculative and warrant further investigation.

The preservation of perceived exertion despite lower blood lactate concentrations and average heart rate during the controlled breathing intervention may have important practical implications. Previous research has demonstrated that controlled breathing interventions can favorably influence autonomic regulation, stress, emotional state, and cardiorespiratory function (Jerath et al., 2006; Zaccaro et al., 2018; Laborde et al., 2022). However, relatively few investigations have examined controlled breathing during dynamic exercise. Among the limited available studies, Fabre et al. (2007a) reported that paced breathing during submaximal roller ski exercise reduced ratings of perceived exertion without altering oxygen uptake, suggesting that breathing strategies can influence subjective exercise perception independently of whole-body metabolic demand (Paced breathing in roller-ski skating: Effects on metabolic rate and poling forces, 2007). Fabre et al. (2007b) further demonstrated that synchronization of breathing with body movement during exercise is influenced predominantly by neuromechanical interactions rather than metabolic stimuli alone, supporting the concept that coordinated breathing and movement represent important physiological characteristics of exercise (Fabre et al., 2007).

The present findings differ from those reported by Fabre and colleagues. Whereas paced breathing in their study reduced perceived exertion, the standardized breathing intervention employed in the present investigation lowered blood lactate concentrations and average heart rate without altering ratings of perceived exertion (Paced breathing in roller-ski skating: Effects on metabolic rate and poling forces, 2007). These differing observations suggest that distinct controlled breathing strategies may influence physiological and perceptual responses through different mechanisms depending on the breathing pattern employed, the exercise modality, and the overall structure of the intervention. Collectively, the available evidence indicates that standardized breathing interventions may modify physiological responses to exercise without necessarily producing parallel changes in perceived exertion. This apparent dissociation between physiological strain and subjective effort further supports the concept that exercise perception emerges from the integration of multiple interacting cardiovascular, respiratory, metabolic, neuromuscular, and cognitive processes rather than from blood lactate concentration or cardiovascular responses alone. Future investigations incorporating direct measurements of respiratory effort, ventilatory parameters, respiratory muscle activity, respiratory gas exchange, end-tidal carbon dioxide, dyspnea, affective responses, autonomic function, central motor drive, and arterial blood gases will be necessary to better understand the complex relationship between controlled breathing, physiological strain, and perceived exertion.

### 4.5 Limitations and future directions

Several limitations should be considered when interpreting the present findings. First, although the within subject crossover design minimized interindividual variability by allowing each participant to serve as their own control, the interventions were completed in a fixed order rather than randomized. This design was intentionally selected to minimize potential learning effects associated with the highly standardized controlled breathing intervention. Because participants were required to learn a specific breathing pattern consisting of prescribed inhalation timing, prolonged exhalation, timed end expiratory breath holds, and synchronized breathing with movement, randomizing the intervention order could have increased the likelihood that participants would unintentionally incorporate elements of the breathing strategy during subsequent control or normobaric hypoxic sessions. Nevertheless, a fixed intervention sequence introduces the possibility of order or familiarization effects that cannot be completely excluded (Senn, 2002). Although exercise workload, breathing cadence, movement cadence, testing procedures, and time of day were carefully standardized across all visits, and sessions were separated by at least 48 hours, future investigations incorporating formal familiarization sessions together with randomized intervention order would further strengthen the experimental design.

Second, the normobaric hypoxic intervention required participants to wear a hypoxic delivery mask. Although this approach is commonly used in exercise research, the mask itself may have influenced breathing comfort, respiratory mechanics, thermal sensation, and the subjective experience of exercise independently of the reduced inspired oxygen concentration. Consequently, it is not possible to completely separate the physiological effects of normobaric hypoxia from the perceptual or mechanical effects associated with wearing the mask. Future studies using simulated altitude rooms or environmental hypoxic chambers could provide an important methodological advantage by exposing participants to normobaric hypoxia without the potential confounding influence of a face mask. Sham mask control conditions may also help distinguish mask related effects from those attributable to hypoxia itself.

Third, although the present study demonstrated clear physiological differences between interventions, it was not designed to identify the mechanisms responsible for these responses. Several physiological variables that may plausibly influence blood lactate regulation during controlled breathing were not directly assessed, including respiratory gas exchange, end tidal carbon dioxide, arterial blood gases, respiratory muscle activity, respiratory muscle oxygenation, indirect calorimetry, catecholamine concentrations, autonomic function, respiratory mechanics, breathing movement synchronization, and respiratory muscle electromyography. Consequently, the physiological explanations discussed throughout this manuscript should be considered hypothesis generating rather than mechanistic conclusions. Future investigations incorporating these measurements will be essential for determining the mechanisms through which controlled breathing influences exercise metabolism.

Fourth, peripheral oxygen saturation was measured using pulse oximetry, which estimates arterial hemoglobin oxygen saturation but does not directly assess skeletal muscle oxygenation, tissue oxygen extraction, local oxygen utilization, regional blood flow, or the temporal fluctuations in oxygen availability that may occur during intermittent breath hold exercise (Ferrari et al., 2011; Perrey and Ferrari, 2018). Accordingly, similar average peripheral oxygen saturation values should not be interpreted as evidence of equivalent tissue oxygenation or metabolic conditions during exercise. Future studies combining pulse oximetry with near infrared spectroscopy, respiratory gas analysis, arterial blood gases, indirect calorimetry, respiratory muscle assessments, and blood gas measurements would provide a more comprehensive assessment of oxygen transport, ventilatory physiology, and tissue oxygen utilization during controlled breathing exercise.

Finally, participants consisted of healthy, recreationally active young adults, which may limit the generalizability of the findings to other populations, including older adults, sedentary individuals, highly trained athletes, and individuals with cardiopulmonary or metabolic disease. In addition, the controlled breathing intervention represented a single standardized breathing strategy. Whether similar physiological responses would be observed using different breathing protocols, exercise modalities, or exercise intensities remains unknown.

Despite these limitations, the present study possesses several important strengths. The within-subject crossover design substantially reduced between-subject variability while allowing direct comparison of three carefully standardized experimental conditions. Exercise duration, movement sequence, external workload, breathing cadence, and movement cadence were synchronized using a standardized 60 beats·min^−1^ metronome to ensure consistent timing across all exercise sessions, while verbal instruction, physiological measurement timing, and testing procedures were standardized across all conditions. Importantly, the controlled breathing intervention incorporated a highly standardized breathing protocol consisting of a double staccato nasal inhalation synchronized to the metronome, followed by a prolonged expiratory phase divided into four sequential auditory cues (“blow,” “hiss,” “soft shh,” and “loud shh”) and a timed end-expiratory breath hold. These auditory cues standardized the temporal structure and behavioral execution of the breathing cycle while providing an audible method for monitoring participant adherence throughout the intervention. Because substantial inter-individual variability exists in the timing and pattern of spontaneous breathing (Tobin et al., 1988), this structured breathing protocol represents a reproducible methodological framework that may facilitate greater consistency across participants and future investigations. The inclusion of both a normobaric hypoxic intervention and a standardized controlled breathing intervention enabled direct comparison of two distinct experimental approaches that produced similar reductions in peripheral oxygen saturation during exercise, allowing differences in metabolic responses to be evaluated under carefully controlled conditions. Repeated measurements of blood lactate, continuous monitoring of peripheral oxygen saturation and heart rate, and analysis using contemporary linear mixed-effects models further strengthened the physiological interpretation and statistical rigor of the study.

The present findings identify several important directions for future research. Studies incorporating direct measures of skeletal muscle oxygenation, respiratory gas exchange, end tidal carbon dioxide, arterial blood gases, respiratory muscle electromyography, respiratory muscle oxygenation, indirect calorimetry, catecholamine responses, autonomic function, respiratory mechanics, and breathing movement synchronization will be essential for clarifying the physiological mechanisms underlying the lower blood lactate concentrations observed during the controlled breathing intervention. Particular attention should be directed toward determining whether differences in the metabolic cost of ventilation, respiratory muscle recruitment, and breathing movement synchronization contribute to the lower heart rate and blood lactate concentrations observed despite comparable external workloads. Future investigations should also evaluate different standardized breathing strategies across a broader range of exercise intensities, exercise modalities, and athletic and clinical populations. Collectively, the present study establishes a rigorous experimental framework and provides a strong foundation for future mechanistic investigations examining how standardized breathing interventions influence oxygenation, respiratory physiology, cardiovascular regulation, and exercise metabolism.

## 5 Conclusion

The present exploratory crossover study demonstrated that a standardized controlled breathing intervention was associated with lower blood lactate concentrations during moderate intensity exercise than both normobaric hypoxic exercise and control exercise. In addition, the controlled breathing intervention was associated with a lower average heart rate while maintaining similar ratings of perceived exertion. Together, these findings were contrary to our original hypothesis and suggest that pulse oximetry measurements alone may not fully explain metabolic responses during moderate intensity exercise. These findings further suggest that physiological factors beyond oxygen availability, including ventilatory physiology, respiratory muscle energetics, autonomic regulation, and breathing movement synchronization, may contribute to the metabolic responses observed during controlled breathing exercise (Dempsey et al., 2006; Fabre et al., 2007; Brooks, 2018).

Because the present study did not directly assess skeletal muscle oxygenation, respiratory gas exchange, arterial blood gases, end tidal carbon dioxide, respiratory muscle activity, respiratory mechanics, autonomic function, catecholamine responses, or breathing movement synchronization, the physiological mechanisms underlying these responses remain unknown. Accordingly, the present findings should be interpreted as identifying an unexpected physiological response that warrants further mechanistic investigation rather than establishing a definitive physiological mechanism.

Future randomized studies incorporating direct assessments of skeletal muscle oxygenation, respiratory gas exchange, arterial blood gases, end tidal carbon dioxide, respiratory muscle electromyography, respiratory muscle oxygenation, indirect calorimetry, autonomic function, catecholamine responses, respiratory mechanics, and breathing movement synchronization are needed to determine the mechanisms underlying these findings and to establish whether similar responses occur across different exercise modalities, exercise intensities, and populations.

Collectively, the present findings demonstrate that standardized controlled breathing can alter metabolic and cardiovascular responses to moderate intensity exercise independent of changes in peripheral oxygen saturation measured by pulse oximetry. By identifying a dissociation between peripheral oxygen saturation, blood lactate concentration, heart rate, and perceived exertion, this study provides a foundation for future mechanistic investigations examining how controlled breathing influences exercise physiology and metabolic regulation.

## Data Availability

The original contributions presented in the study are included in the article/supplementary material. Further inquiries can be directed to the corresponding author.
